# Sänger’s Cæsarean Operation

**Published:** 1886-08

**Authors:** 


### *SÄNGER'S CÆSAREAN OPERATION.*

At a meeting of the Philadelphia Obstetrical Society, held May 6, 1886, Dr. Robert P. Harris reported the contents of a letter from Dr. Sänger, of Leipzig, relative to the success which has attended the modified Cæsarean operation bearing his name. The Porro operation, as practiced at the Santo Cateima Hospital, of Milan, Italy, has until recently held a position in advan[illegible] of any Cæsarean operation in the percentage of recoveries; now Sänger's stands at the head. Dr. Sänger reports that his operation has been done twenty-five times, saving eighteen women, or 72 per cent., and twenty-two children, or 88 per cent. Of these, three were performed in America and were fatal. These American cases were hopeless, because of delay in their performance.

The twenty-two operations done in Europe saved eighteen women, or 81 9-11 per cent. Dr. Sänger believes this operation should be preferred to craniotomy, because of its moderate fatality, and its saving the child. Dr. Harris said: "We should be glad if all of the Cæsarean operations of the United States should be performed after Sänger's method, as simplified by Garrigues and Leopold; but we must not expect very happy results here until our accoucheurs become alive to the fact that delay in operating will make any method fatal in a large proportion of cases. In no country are the capabilities of the old Cæsarean operation greater than in the United States, and in few has this form of delivery been of late more uniformly fatal. To find eighteen recoveries under it, we must search backward to January, 1863, and through a record of time covering more than twenty-three years, in which period seventy-three operations have been performed proving fatal about 75 per cent. This occurred notwithstanding an established fact that a set of early operations will save 75 per cent. of the women, and still higher of the children, in the United States.

